# Rating Down for Publication Bias is Rare in Cochrane Reviews: A Descriptive Study

**DOI:** 10.1002/cesm.70089

**Published:** 2026-07-21

**Authors:** Markus Toews, Silvia Minozzi, Bernardine H. Stegeman, Michela Cinquini, Joerg J. Meerpohl, Kenneth Färnqvist, Matteo Bruschettini, Ingrid Toews

**Affiliations:** ^1^ Institute for Evidence in Medicine, Medical Center—University of Freiburg, Faculty of Medicine University of Freiburg Freiburg Germany; ^2^ Laboratory of Methodology of Systematic Reviews and Guidelines Production Istituto di Ricerche Farmacologiche Mario Negri IRCCS Milan Italy; ^3^ Knowledge Institute of the Dutch Association of Medical Specialists Utrecht the Netherlands; ^4^ Cochrane Germany, Cochrane Germany Foundation Freiburg Germany; ^5^ Department of Molecular Medicine and Surgery Karolinska Institute Solna Sweden; ^6^ Cochrane Sweden, Department of Research, Development, Education and Innovation, Skåne University Hospital Lund University Lund Sweden

**Keywords:** dissemination bias, GRADE, meta‐research, publication bias, research on research, systematic reviews

## Abstract

**Background:**

Publication bias impacts the direction and magnitude of summary effect estimates in systematic reviews and the confidence in the review findings. It remains unclear to what degree publication bias is considered in the certainty assessments in systematic reviews. Our research aims are to generate an overview of and estimate how frequent rating down occurs for the GRADE domain “publication bias” across Cochrane reviews.

**Study Design and Setting:**

This is a descriptive study. We included Cochrane reviews published between January 2021 and October 2023 that included at least one outcome for which the certainty of evidence was rated down for publication bias. We collected data from the reviews' full text, analyses, and figures. We analyzed data with descriptive statistical analysis. We used a deductive qualitative approach for analyzing qualitative data.

**Results:**

We identified 81 reviews. These reviews included between 1 and 328 individual studies. All but three reviews included randomized‐controlled trials only. Data from less than 10 studies supported the summary of findings of 106 outcomes. The most commonly used methods for detecting publication bias were funnel plots. 220 outcomes were rated down by one level for publication bias. Four outcomes were rated down by two levels. The most commonly reported reasons were indications of publication bias in funnel plots.

**Conclusions:**

Rating down for publication bias was rare in Cochrane reviews despite the high prevalence of publication bias in original research. Guidance might not suffice to inform assessments of publication bias in reviews with less than 10 studies.

## Summary

**What is Already Known?:**

- Publication bias occurs when studies with significant or positive results are more likely to be published than those with non‐significant or negative results.
- Publication bias impacts on the direction and magnitude of summary effect estimates in systematic reviews and the confidence in the review findings.

**What is New?:**

- Less than 8% of Cochrane Reviews rated down for publication bias according to GRADE.
- The most commonly used method for assessing publication bias were funnel plots.
- In four instances the authors rated down the certainty of evidence by 2 steps for publication bias.

**Potential Impact:**

- Available guidance might not suffice to optimally inform assessments of publication bias in reviews with less than 10 studies.
- Practical and unambiguous guidance is needed for assessing publication bias in systematic reviews and GRADE assessments.

## 1 Background

With around 50% of the scientific evidence remaining unpublished [1], bias due to missing evidence, often called publication bias, dissemination bias, reporting bias or non‐reporting bias, is generally observed to be impacting various research fields and topics in health and healthcare [2, 3, 4]. Bias due to missing evidence is a systematic error impacting the direction and magnitude of summary effect estimates or synthesized results in systematic reviews. This potential flaw is reflected in the (Grading of Recommendations Assessment, Development, and Evaluation) GRADE approach as “publication bias” under the domain “other considerations” for rating the certainty of the evidence [5]. GRADE is an approach for rating the certainty of evidence in systematic reviews of health interventions and provides explicit criteria for rating the certainty of the evidence that include study design, risk of bias, imprecision, inconsistency, indirectness, and publication bias [5].

In some cases, evidence of bias due to missing evidence clearly shows an impact on a body of evidence; however, the assessment of the presence and impact of this bias is more challenging in other bodies of evidence, topics, and research fields. Challenges often occur when the body of evidence is small and no meaningful funnel plots can be constructed, for example, when less than 10 studies are included [6]. This threshold is based on a rule of thumb as per the Cochrane Handbook [6]. Moreover, when the fate of studies can be traced less completely, such as in non‐registered, non‐randomized studies, non‐pharmacological and public health interventions, publication bias and its impact are more difficult to assess.

There are a number of developments and observations that can aid an accurate assessment of bias due to missing evidence [7]. Methodological approaches for assessing this bias are advancing continuously. This form of bias is often assessed on the basis of funnel plots. Within funnel plots, asymmetry indicates small study effects, which can potentially be a result of publication bias. Acknowledging the limitations of simple funnel plots [8] and the low statistical power of other tests [6], advanced statistical methods and systematically developed frameworks have been shown to capture the issue in greater detail and precision, taking information from different sources into account. These approaches include, among others, the use of contour‐enhanced funnel plots, the use of the Risk of Bias due to Missing Evidence (RoB‐ME) tool [9] and the Risk of Bias 2 tool [10] that disentangle review‐level bias (missing evidence) from study‐related biases (selective reporting) and thus support a clearer assessment of bias due to missing evidence. Besides graphical—and risk of bias tools, methods for the assessment of publication bias are advancing, covering various selection models [11], van Assen et al. [12, 13], and other statistical methods [7].

While there is clear evidence on the extent of bias due to missing evidence in original research [2, 14], it remains unclear to what degree the systematic non‐dissemination of research is detected in and finds practical consideration in the assessments of certainty of evidence in systematic reviews. With this research, we want to capture the current assessments of the GRADE domain “publication bias” and subsequent decisions about grading the evidence. Therefore, our research aims are:

1. to (a) generate an overview of and (b) estimate how frequently rating down occurs for the GRADE domain “publication bias” across Cochrane reviews of interventions;
2. to build a “knowledge base” of reasons for downgrading the certainty of the evidence due to publication bias and to locate examples where downgrading by one or two steps was done.

## 2 Methods

This study follows the design of a cross‐sectional study. It is reported according to the Strengthening the Reporting of Observational Studies in Epidemiology (STROBE) Statement [15].

### 2.1 Sampling and Eligibility Criteria

We included all Cochrane reviews that investigated the effectiveness of interventions, including those with network‐ and pairwise meta‐analyses that were published between January 2021 and October 2023. We selected this period to account for the publication of the Cochrane Handbook for Systematic Reviews of Interventions [16] and its potential implementation in the review process. Eligible reviews had to include at least one outcome for which the certainty of evidence was rated down for publication bias according to the GRADE approach.

We excluded reviews with no included studies, qualitative evidence synthesis, rapid reviews, scoping reviews, overviews of reviews, systematic reviews of diagnostic accuracy, prognostic reviews, and methodological reviews.

### 2.2 Literature Searches and Review Selection

Potentially eligible reviews were retrieved from the Cochrane Database of Systematic Reviews via the Cochrane Library. We searched the Cochrane Library on 25th October 2023 for reviews published from 01 January 2021 until October 2023 (M.T.). We established a transparent and reproducible natural language processing (NLP) pipeline throughout our workflow. First, we generated the URLs (links) for all reviews to access the review information (M.T.). For each URL, the web page was semi‐automatically accessed, and the textual content was extracted from the paragraph elements, including the summary of findings and especially the footnotes of the GRADEing (M.T.). This was achieved using web scraping tools capable of parsing HTML content.

Leveraging web scraping and text mining techniques in R 4.3.2 [17] using the rvest package (v1.0.4) [18], we extracted and examined relevant sentences based on predefined text sections and phrases related to publication bias (M.T.). All text was converted to lowercase for case‐insensitive matching, in various spellings and, if applicable, with truncated words.

To comprehensively identify mentions of publication bias and its assessment aiding review selection, we used an iterative approach where irrelevant text words were discarded and potentially relevant text words were added to increase the sensitivity of our NLP pipeline. This means that we used an initial set of text words and added new meaningful text words to the existing list when we identified them in the explanations to summary of findings tables or the respective methods section. Potentially relevant text words were identified from all retrieved reviews. These five thematic groups systematically captured direct references to publication bias, (“publication bias,” “publication‐bias,” “publication–bias,” “dissemination bias,” “other considerations”) AND (“downgrad,” “rated”). By organizing filter terms in this structured way, we enhanced the sensitivity and thoroughness of our automated text extraction across diverse reporting styles and terminology in the retrieved reviews.

Textual data containing the relevant text words was extracted automatically from RStudio to MS Excel for subsequent review (M.T.). Two reviewers independently assessed the eligibility of each review based on the reporting of publication bias assessment in this automatically extracted textual data (M.T. and I.T.). One reviewer also confirmed that the filtered‐out reviews were correctly discarded from the sample (M.T.). The full reproducible code for the NLP pipeline, including all steps and parameters, will be made available open access via OSF.

### 2.3 Data Collection

We collected data from the reviews' full text, analyses and figures in a tailored and piloted data extraction sheet in MS Excel (M.T., S.M., B.H.S., M.C., K.F., M.B., and I.T.).

### 2.4 Outcomes

The categories for data extraction included:

- general information about the review (e.g., title, topic, year of publication, number of included studies and participants etc.);
- details about the review topic and methods (e.g., pharmacological review, risk of bias tool used);
- information about the outcome of interest for our study (e.g., outcome name, effect estimate, number of included studies and participants);
- details about methods for assessing publication bias (use of funnel plots, use of RoB‐ME);
- rating of publication bias for each outcome of interest.

Review titles were mainly extracted for unambiguous identification of the record and not used in the analyses.

Data were partially extracted automatically by text mining and web scraping approaches in RStudio. We used a NLP pipeline for textual data extraction. The information regarding title, topic, and year of publication was extracted fully automatically. For a semi‐automated extraction, we defined the following distinct groups of filter terms within our NLP pipeline: (1) direct references to publication bias, (“publication bias”, “publication‐bias,” “publication–bias,” “dissemination bias”) AND (“downgrad,” “rated”), (2) key methods of bias detection (“funnel plots,” “small‐study effect,” “small‐study,” “small study,” “contour enhanced”, “contour‐enhanced,” “contour‐enhanced,” “asymmetry,” “symmetric,” “skew,” “page 2019,” “page 2020,” “page 2022,” “risk of bias due to missing evidence,” “missing evidence,” “rob‐me”), (3) established risk of bias tools (“risk of bias 1,” “rob1,” “rob 1,” “risk of bias tool 1,” “risk of bias assessment tool,” “risk of bias tool,” “higgins 2011,” “higgins 2017,” “higgins 2019,” “risk of bias 2,” “rob2,” “rob 2,” “risk of bias tool 2”), (4) sources of potential funding or industry‐related bias (“fund,” “industr,” “funding source”) and (5) broader terminology for publication bias (“other consideration”).

Relevant text segments containing the above predefined terms were automatically compiled verbatim into an MS Excel template for further evaluation. One author (M.T., S.M., B.H.S., M.C., K.F., M.B., and I.T.) reviewed these segments for relevant information and coded the information, respectively, with discrepancies resolved through discussion. Another reviewer checked and validated all extracted and coded data.

Data for all other outcomes were extracted by one author and cross‐checked by a second independent author. If we had encountered missing data, we would have contacted the review authors.

### 2.5 Data Analysis

We analyzed all quantitative data with descriptive statistical analysis (M.T. and I.T.). All findings are reported as absolute numbers and proportions or medians and their ranges. General information about the reviews was analyzed descriptively to characterize our sample. Moreover, we used information on the review topic and outcome type to categorize the reviews and describe our sample.

We used a deductive qualitative approach for analyzing qualitative data, that is, reasons reported by review authors why a body of evidence was rated down for publication bias (I.T.). Such qualitative findings were reported narratively.

**Figure 1:**
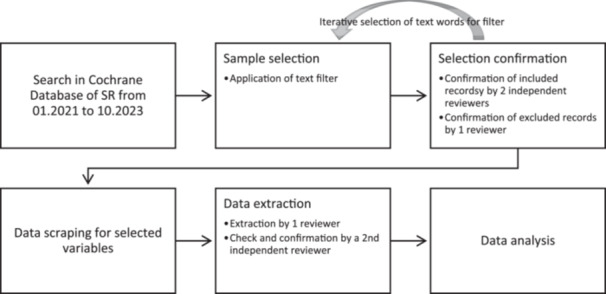
Flow of methods.

A summary of the methods used is shown in Figure 1.

## 3 Results

**Figure 2:**
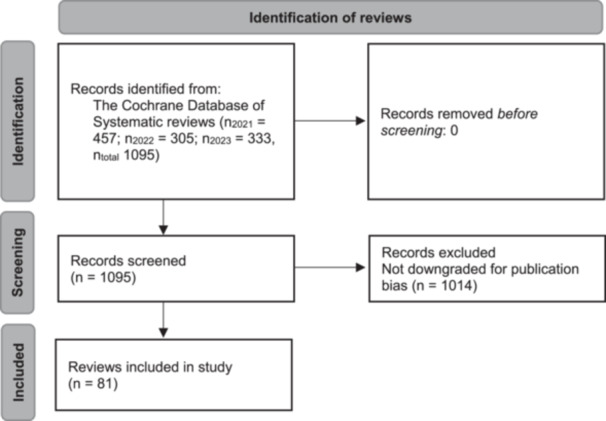
Flow chart of included reviews.

Out of 1095 reviews, we included 81 reviews, including 3 network meta‐analyses. We excluded 1014 reviews because they did not report a downgrade for any outcome in their GRADE summary of findings tables (see Figure 2). Accordingly, 7.4% of all Cochrane Reviews of Interventions rated down at least one outcome for “publication bias” in their GRADE assessment.

## 4 Description of Sample

Main review and outcome characteristics are listed in Table 1 and all references are included in Supporting Information S1: 1. Seventy‐eight reviews included RCTs (randomized controlled trials) only and used the corresponding risk of bias tool. The RoB‐ME tool [9] was used in none of the included reviews, likely because the tool was only published in November 2023. The included reviews were published by a variety of review groups (see Supporting Information S1: Table 1) and investigated a wide range of comparisons and outcomes (see Supporting Information S1: Table 2). The main outcome categories were: mortality and survival outcomes, adverse events and safety, quality of life, maternal and neonatal outcomes, procedure‐ and surgery‐related outcomes, cardiovascular and thromboembolic outcomes, and health system, economic and utilization outcomes.

**Table 1:** Characteristics of included reviews and outcomes of interest.

	2021	2022	2023	Total
*Review characteristics*				
Number of reviews included/Total number of reviews published	35/457	24 (incl. 1 NMA)/305	22 (incl. 2 NMAs)/333	81
Median number of included studies (range)	19 (2 to 249)	11 (2 to 222)	34 (1 to 328)	19 (1 to 328)
Median population size by review (range)	3972 (321 to 178,813)	1696 (218 to 189,283)	3361 (11 to 47,106)	3182 (11 to 178,813)
Intervention under investigation	Pharmacological 18 (51.4%) Non‐pharmacological 15 (42.9%) Mixed 2 (%5.7)	Pharmacological 12 (50.00%) Non‐pharmacological 10 (41.67%) Mixed 2 (8.33%)	Pharmacological 9 (40.91%) Non‐pharmacological 10 (45.45%) Mixed 3 (13.64%)	Pharmacological 39 (48.15%) Non‐pharmacological 35 (43.21%) Mixed 7 (8.64%)
RoB tool used	RoB 1 26 (74.28%) RoB 2 5 (14.29%) ROBINS‐I^$^ 2 (5.71%)* 1 (2.86%) Other 1 (2.86%)	RoB 1 21 (87.5%) RoB 2 2 (8.33%) ROBINS‐I 1 (4.17%)	RoB 1 19 (86.36%) RoB 2 3 (13.64%)	RoB 1 66 (81.48%) RoB 2 10 (12.35%) ROBINS‐I 3 (3.71%) Mixed 1 (1.23%) Other 1 (1.23%)
*Outcome characteristics*				
Number of outcomes included	111	58	65	234
Median number of included studies (range)	4 (1 to 64)	4 (1 to 47)	4 (1 to 190)	4 (1 to 190)
Median population size per included outcome (range)	688.5 (27 to 13,341)	495 (30 to 20,521)	431 (9 to 24,817)	591.5 (9 to 24,817)
Type of included studies	RCTs 109 (98.20%) Non‐RCTs 2 (1.80%)	RCTs 57 (98.28%) Non‐RCTs 1 (1.72%)	RCTs 65 (100%)	RCTs 231 (98.72%) Non‐RCTs 3 (1.28%)
Effect estimate statistically significant	Yes 48 (43.24%) No 39 (35.14%) Not applicable^$^ 24 (21.62%)	Yes 22 (37.93%) No 27 (46.55%) Not applicable^$^ 9 (15.52%)	Yes 25 (38.46%) No 29 (44.62%) Not applicable^$^ 11 (16.92%)	Yes 95 (40.60%) No 95 (40.60%) Not applicable^$^ 44 (18.80%)
Funnel plot created	Yes 31 (27.93%) No 80 (72.07%)	Yes 22 (37.93%) No 36 (62.07%)	Yes 27 (41.54%) No 35 (53.85%) Unclear 3 (4.62%)^b^	Yes 80 (34.19%) No 151 (64.53%) Unclear 3 (1.28%)^b^
Certainty of evidence for the outcome of interest	Moderate 7 (6.31%) Low 33 (29.73%) Very low 71 (63.96%)	Moderate 1 (1.72%) Low 22 (37.93%) Very low 35 (60.35%)	Moderate 6 (9.23%) Low 24 (36.92%) Very low 35 (53.85%)	Moderate 14 (5.98%) Low 79 (33.76%) Very low 141 (60.26%)

*Note:* $ for example in narratively reported outcomes; NMA = network meta‐analysis; ^$^See: Sterne et al. “ROBINS‐I: a tool for assessing risk of bias in non‐randomized studies of interventions.” BMJ 355: i4919.

a Authors used Rob 2, RoB 1 was used in previously included studies;

b Authors report that funnel plots would be created in methods section, but results or figures include no findings about funnel plots or plots themselves.

**Table 2:** Overview of levels for downgrading for publication bias in GRADE assessments.

	2021	2022	2023	Total
Levels of downgrading for publication bias	1 level 101 (90.99%) 2 levels 1 (0.90%) Other^a^ 9 (8.11%)	1 level 58 (100%)	1 level 61 (93.85%) 2 levels 3 (4.62%) Other^a^ 1 (1.53%)	1 level 220 (94.02%) 2 levels 4 (1.71%) Other^a^ 10 (4.27%)

a see narrative description of findings: four not rated down; six rated down by half a level.

The effect estimates for the included outcomes were rather evenly distributed to be statistically significant and statistically not significant. For the majority of outcomes, the review authors had not created funnel plots. The certainty of evidence was mainly very low and low, with only very few outcomes (*n* = 14; 5.98%) having moderate certainty of evidence.

## 5 Ratings of Publication Bias

Ratings for publication bias are reported in Table 2. There was a total of four outcomes in three reviews that were rated down by two levels for publication bias. The author's basis for rating down was the unavailability of study data in full scientific publications, in one review with network meta‐analysis (the other two network meta‐analyses rated down by one). In another review, the authors simply stated that they rated down by two levels for suspected publication bias. In another review, the authors combined their rating for publication bias with a rating for other biases, and this combined rating resulted in a rating down by two levels. In another review, authors suspected publication bias but did not rate it down because the certainty was already very low. In another review, the authors did not rate down despite suspected publication bias because they suspected that publication bias caused heterogeneity and, hence, rated down for inconsistency. Then, there was one review with six outcomes where the authors rated down by half a level for publication bias.

## 6 Reasons for Rating Down for Publication Bias

In the explanations to the reviews' Summary of Findings tables, the review authors documented various reasons for rating down the certainty of the evidence due to publication bias. The reasons are listed in Table 3. Rarely, review authors reported multiple reasons for downgrading, for example, funnel plot asymmetry and results of the Egger's test, therefore, the numbers do not add up to the total of 234 included outcomes. One outcome was not rated down for publication bias, as the certainty of evidence was already very low.

**Table 3:** Overview of reasons for downgrading for publication bias in GRADE assessments.

Reason for rating down as per SoF	*N* outcomes that were rated down
Indication of publication bias in funnel plot	67
Selective data missing (reporting bias)	46
Limited availability of studies reporting outcome(s) of relevance	26
Included studies were mainly funded by for‐profit organizations (industry)	12
Suspicion of duplicate publication	5
Based on the results of Egger's test	4
Studies or individual findings not published in a *scientific* publication	2
Only small studies were available	2
Unspecified evidence of publication bias	2
Lack of ability of to test for publication bias	2^b^
Publication bias could not be excluded	1
Based on the results of Moore's test	1
No specific reason reported	63^a^

^a^ For 16 of these outcomes, the authors described in the results section that they detected funnel plot asymmetry but not in the explanations of the summary of findings tables; for two outcomes, the authors reported elsewhere that they had rated down for publication bias because there were no full, peer‐reviewed publications of the results. For 45 outcomes, neither the results section nor the explanations to the summary of findings tables did further specify any reasons for rating down for publication bias;

^b^ The authors report: “We downgraded two levels for high overall risk of bias […] and lack of ability to check for publication bias.”

## 7 Most Common Methods Used to Assess Publication Bias

The most commonly used methods for detecting publication bias, as described in the methods sections of the reviews, were funnel plots only (*n* = 47) or funnel plots in combination with one or more subsequent statistical analyses or tests (*n* = 28). Among these tests, Egger's test (*n* = 12), Harbord's test (*n* = 3), the trim and fill method (*n* = 2) or other unnamed analyses or tests (*n* = 6) were most commonly described. The median number of included studies in reviews that reported using funnel plots was 12 (range 2−190). Although many review authors planned to use funnel plots, only around one‐third of the inspected outcomes had evidence from funnel plots to support the assessment of publication bias (see Table 1). In three reviews (4 outcomes), the authors had created contour‐enhanced funnel plots.

**Figure 3:**
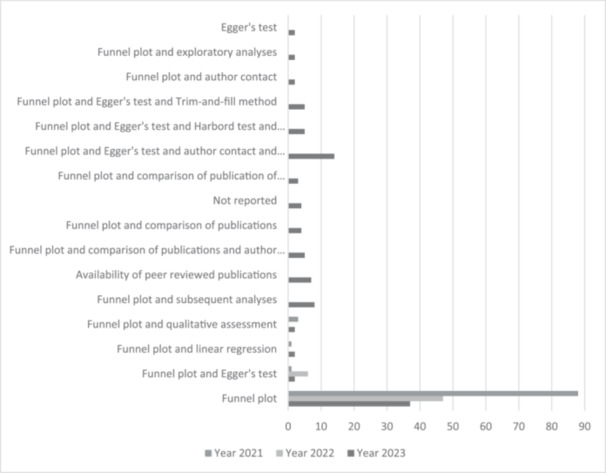
Summary of tools used for the assessment of publication bias by year.

Of all included reviews, authors of five reviews reported to have compared the study publications with the respective database records in a trial registry or study protocols, four author teams followed up the study authors about the availability of study publications or publications of data of individual outcomes. Others used observations specific to their area of research or a qualitative and content‐focused approach to assess the plausibility of publication bias (*n* = 4). For example, in one review, the authors checked for the availability of data on adverse events in publications other than gray literature and conference abstracts. Others judged the unavailability of peer‐reviewed publication as an indicator of publication bias. Lastly, one review assessed whether the included studies were likely to be representative of all relevant studies that have been conducted. Single reviews used one of the following approaches: an assessment of the availability of peer‐reviewed publications, a discussion of the plausibility of publication bias amongst co‐authors, or the ORBIT I and II tool. Five review authors used other tests without using funnel plots. Instead, they used Egger's test, Moore's test, compared the study publications with their study protocols, contacted study authors for unpublished data, or used the availability of peer‐reviewed publications as an indicator for publication bias. The methods were not reported in one review (see Figure 3).

In two of three network meta‐analyses, the authors reported to use comparison adjusted funnel plots to assess publication bias. One network meta‐analysis reported to use funnel plots to assess publication bias.

## 8 Discussion

### 8.1 Summary of Main Findings

We wanted to capture the frequency and underlying reasons for downgrading for publication bias according to GRADE in recently published Cochrane reviews. We included 81 reviews with 234 outcomes that were rated down for publication bias. The included reviews and outcomes varied in the number of included studies as well as their topical focus. The clear majority included RCTs only and used the Cochrane Risk of Bias 1 [19] tool to assess the study quality. Within the included sample, most reviews described to have used funnel plots to assess publication bias and rated down the certainty of the evidence by 1 for publication bias.

### 8.2 Appropriate Use of GRADE Approach/Guidance

Our study shows that rating down for publication bias in Cochrane reviews is rare, despite evidence that 30%–60% of studies remain unpublished [1, 2, 4, 14]. The lack of full availability of research findings is not adequately reflected at the review level, suggesting that more attention to publication bias is needed. Current Cochrane and GRADE guidance include methods such as funnel plots for reviews with ≥10 studies, or study size, funding, novelty, and search limitations for smaller reviews [5]. However, these recommendations are general and inconsistently applied, leading to heterogeneous and often ad hoc justifications for downgrading.

In addition, available approaches, such as funnel plots, have limitations and primarily serve as sensitivity analyses rather than definitive tests. Asymmetries in funnel plots can arise from reasons unrelated to publication bias. Lin and Chu [20] demonstrate that standard funnel plot symmetry tests suffer from low power to detect publication bias and inflated type I error rates, especially under common meta‐analytic conditions like heterogeneity, leading to unreliable inferences. Furuya‐Kanamori et al. Furuya‐Kanamori et al. [21] show these P‐value‐driven methods are underpowered, often failing to identify bias even when present, which can result in overconfident meta‐analytic conclusions. Almalik et al. [22] highlight unreliability due to heteroscedasticity (varying study variances), causing false positives and misattribution of asymmetry to bias rather than legitimate statistical artifacts. Relying on these tests only can harm evidence synthesis by suggesting unwarranted downgrades in GRADE (e.g., for apparent “bias” from small‐study effects) or overlooking true bias, potentially misleading decision‐making. Overall, such overreliance exacerbates errors in systematic reviews, as visual and statistical funnel plot interpretations might be inaccurate. This reinforces our view that while existing methods provide valuable indications, they must be interpreted with caution and contextual understanding rather than applied mechanistically.

For example, Cochrane guidance [6] is of a rather general nature and often difficult to implement, so that assessments based on such guidance could be heterogeneous. This heterogeneity is clearly reflected in our findings, where review authors used various—often ad hoc—rationales for downgrading.

In GRADE Summary of Findings tables, authors drew on a variety of approaches, but funnel plot asymmetry was most frequently cited—although only one‐third of downgrades were supported by funnel plots. Despite their limitations, funnel plots remain the most commonly used tool. This reliance persists even though such methods lack power in reviews with fewer studies and may lead to misleading conclusions. The continued dominance of funnel plots likely stems from their visibility in guidance documents and familiarity among authors, even if their appropriateness is questionable in many review settings. Moreover, study characteristics (i.e., study size) and study funding (i.e., industrial funding) were used as signposts to suspect publication bias. On the other hand, meaningful considerations were often review‐specific and not generalizable. Notably, review authors found that relevant outcomes were not reported in a sufficiently large number of studies, for all (relevant) time points or for all study groups.

Lastly, the reporting was unclear in a large number of reviews, so the rationale of the review authors for rating down remained unclear. Also, the overall reporting of methods used to assess publication bias and the review results was sometimes observed to be incomplete or incoherent, which made a thorough understanding of the reviewers' rationale difficult. In summary, the quality of the assessments for publication bias in GRADE was mixed, and we noted a need for more clarity and coherence in reporting information within reviews to make assessments of publication bias transparent. Furthermore, the variation in how reviews documented their publication bias assessments (e.g., explanation depth in SoF tables, clarity in methodology) limits reproducibility and undermines confidence in GRADE ratings. A standardized, transparent approach would improve the interpretability and utility of evidence summaries.

Clearer guidance and a stronger conceptual understanding are crucial [23]. This clarity is essential to enable a more accurate and honest evaluation of this bias. For instance, in several reviews, the certainty of evidence was downgraded due to the inclusion of studies solely available as conference abstracts. However, it is crucial for authors to consider whether such conference abstracts predominantly present findings that are not fully reported in published studies and what the underlying reasons might be. Simply relying on the unavailability of studies as full publications may not inherently indicate publication bias. This guidance should distinctly differentiate between instances of non‐publication and suspected publication bias, thus enhancing the accuracy and reliability of bias assessment within systematic reviews. On the other hand, noting the various potential reasons for non‐publication [24] and biases that are related to publication bias [25] is crucial. Within the GRADE approach, publication bias is explicitly a domain for downgrading [26]. Clearer emphasis on how reasons for missing studies (e.g., delays or selective non‐submission) should inform nuanced assessments in this domain can aid the accurate assessment.

While our findings illustrate the need for more and clearer guidance for rating publication bias in GRADE, we also acknowledge that rating down for publication bias might be less straightforward and inconsistently applied. Many outcomes were already rated as low or very low certainty for other reasons, which may have limited the influence of an additional downgrade for publication bias on the final certainty rating. This may partly explain why publication bias was seldom rated down, even if it was potentially relevant. Nevertheless, concerns about publication bias should be considered and reported independently of their effect on the overall certainty rating.

### 8.3 Strength and Limitations

The limitations of our study mainly pertain to the sampling approach and the representativeness of our findings. By using a semi‐automated approach with filters for specific combinations of text phrases, we might inadvertently have missed to include reviews and/or outcomes that rated down for publication bias that had not used such phrases. We tried to minimize this risk by checking the textual information that included the phrases “publication bias,” “downgrad” and “other consideration” by two reviewers independently and iteratively refining the search approach, for example, by adding other relevant text phrases. However, we might also have inadvertently missed searching for relevant phrases.

Our sample is likely not representative of systematic reviews in general since we included Cochrane reviews only. Guidance and details in methods for conducting Cochrane and non‐Cochrane reviews might differ, and Cochrane guidance is rather clear about the expected methods for assessing reporting biases and using the GRADE approach. An investigation of non‐Cochrane reviews might have returned different results. Moreover, our sampling period covers the peak of the publication of COVID‐19‐related research evidence. This is reflected in the relatively higher number of reviews and outcomes included in the year 2021. It was an atypical year with regard to publication behavior in health research, which might also have had an impact on our findings. One peer reviewer suggested increasing the sample to cover a larger sample and a post‐pandemic timeframe for additional sampling. However, we respectfully deem our sample large and representative enough to address our study objectives fully. Our sample also did not return rich insights into reviews of evidence from non‐randomized studies. Only three outcomes were supported by a body of evidence from observational studies. In all three instances, the review authors rated down by one step for publication bias (to low or very low certainty of evidence). For all three of these bodies of evidence, the review authors reported to have constructed funnel plots. which indicated asymmetry. One of the reviews also summarized the results of Egger's test that supported the suspicion of publication bias. Based on our observations, we detected no differential assessment of publication bias in randomized and non‐randomized studies in our sample.

Further, we included only reviews that had downgraded the certainty of the evidence for publication bias. Valuable information about the assessment of the evidence in light of publication bias might also have emerged from reviews that did not downgrade for publication bias. Such reviews might be the subject of future research and would offer a more comprehensive evaluation of publication bias in systematic reviews. A complete account of publication bias should also assess when and why the certainty of evidence was not rated down for publication bias. From a methodological perspective on this study, the lack of a registered protocol might pose another limitation to our work.

However, the strength of our review included a systematic approach to our research aims with an internal study protocol in place, ongoing exchange about the research methods, and rigorous piloting of study steps, such as the methods for searching and the data extraction. Drawing the sample by screening retrieved search results in duplicate and independently, as well as automated and human data extraction, has supported the reliability of our approach and findings. Despite the limitations described above, we successfully generated a large and up‐to‐date study sample that is representative of currently published Cochrane reviews. Lastly, the composition of the research team was made up of researchers from various levels with a thorough understanding of Cochrane, GRADE, and publication bias.

### 8.4 Implications for Future Research

One key area for future research lies in refining the (GRADE and Cochrane) guidance for assessing publication bias within systematic reviews and its implementation. This includes more intense exploring of alternative approaches to assessing publication bias that may provide more nuanced insights into the reliability of the evidence base. Another critical area for future research is the development of guidance tailored to systematic reviews that include a limited number of studies, particularly those with fewer than 10 studies. Moreover, there is a need for research into instances where a rating down for publication bias by more than one step might be warranted. Current guidance recommends a single‐step downgrade for suspected publication bias; however, there may be situations where the evidence of bias is particularly strong or where multiple sources of bias are present, necessitating a more substantial adjustment. Future research should aim to identify such scenarios and develop criteria for determining when a 2‐level downgrade is appropriate.

## 9 Conclusions

Rating down for publication bias was relatively rare in Cochrane reviews despite the high prevalence of non‐publication and possible publication bias in original research. The standard guidance might not suffice to inform assessments of publication bias in reviews with less than 10 studies.

## Author Contributions

**Markus Toews:** conceptualization, investigation, methodology, validation, writing – review and editing, data curation, writing – original draft, software. **Silvia Minozzi:** conceptualization, investigation, methodology, validation, writing – review and editing, data curation. **Bernardine H. Stegeman:** investigation, data curation, writing – review and editing, validation, methodology. **Michela Cinquini:** investigation, writing – review and editing, validation, data curation. **Joerg J. Meerpohl:** conceptualization, writing – review and editing, methodology. **Kenneth Färnqvist:** investigation, writing – review and editing, validation, data curation. **Matteo Bruschettini:** conceptualization, investigation, writing – review and editing, validation, methodology, data curation. **Ingrid Toews:** conceptualization, investigation, writing – original draft, methodology, validation, visualization, writing – review and editing, formal analysis, supervision, project administration, data curation, resources, software.

## Funding

The authors have nothing to report.

## Ethics Statement

The authors have nothing to report.

## Conflicts of Interest

M.B., S.M., M.C., J.J.M., B.S., and I.T. are members of the GRADE Working Group and Cochrane. B.S. is a member of the Dutch GRADE Network.

## Supporting information

Supporting File:

## Data Availability

All extracted data and code will be made available upon request to the corresponding author.
