# Supplementary material for: Rating Down for Publication Bias is Rare in Cochrane Reviews: A Descriptive Study
Source: Cochrane Evid Synth Methods. 2026 Jul 21;4(4):e70089. doi: 10.1002/cesm.70089 (PMC13410555; doi:10.1002/cesm.70089)
Supplement: Supplementary file 1 — Supporting File: [file CESM-4-e70089-s001.docx]

Supplement 1. List of included reviews

| **Authors** | **Title** | **Year** | **Issue** | **URL** |
| --- | --- | --- | --- | --- |
| Abbasciano, RG; Tomassini, S; Roman, MA; Rizzello, A; Pathak, S; Ramzi, J; Lucarelli, C; Layton, G; Butt, A; Lai, F; Kumar, T; Wozniak, MJ; Murphy, GJ | Effects of interventions targeting the systemic inflammatory response to cardiac surgery on clinical outcomes in adults | 2023 | 10 | <https://www.cochranelibrary.com/cdsr/doi/10.1002/14651858.CD013584.pub2> |
| Purgato, M; Prina, E; Ceccarelli, C; Cadorin, C; Abdulmalik, JO; Amaddeo, F; Arcari, L; Churchill, R; Jordans, MJD; Lund, C; Papola, D; Uphoff, E; van Ginneken, N; Tol, WAnton; Barbui, C | Primary‐level and community worker interventions for the prevention of mental disorders and the promotion of well‐being in low‐ and middle‐income countries | 2023 | 10 | <https://www.cochranelibrary.com/cdsr/doi/10.1002/14651858.CD014722.pub2> |
| Sharif, S; Meader, N; Oddie, SJ; Rojas-Reyes, MX.; McGuire, W | Probiotics to prevent necrotising enterocolitis in very preterm or very low birth weight infants | 2023 | 7 | <https://www.cochranelibrary.com/cdsr/doi/10.1002/14651858.CD005496.pub6> |
| Hay, S; Ovelman, C; Zupancic, JAF; Doyle, LW; Onland, W; Konstantinidis, M; Shah, PS; Soll, R | Systemic corticosteroids for the prevention of bronchopulmonary dysplasia, a network meta‐analysis | 2023 | 8 | <https://www.cochranelibrary.com/cdsr/doi/10.1002/14651858.CD013730.pub2> |
| Rosser, BA; Fisher, E; Janjua, S; Eccleston, C; Keogh, E; Duggan, G | Psychological therapies delivered remotely for the management of chronic pain (excluding headache) in adults | 2023 | 8 | <https://www.cochranelibrary.com/cdsr/doi/10.1002/14651858.CD013863.pub2> |
| Yoong, SL; Lum, M; Wolfenden, L; Jackson, J; Barnes, C; Hall, AE; McCrabb, S; Pearson, N; Lane, C; Jones, JZ; Nolan, E; Dinour, L; McDonnell, T; Booth, D; Grady, A | Healthy eating interventions delivered in early childhood education and care settings for improving the diet of children aged six months to six years | 2023 | 8 | <https://www.cochranelibrary.com/cdsr/doi/10.1002/14651858.CD013862.pub3> |
| Tighe, MP; Andrews, E; Liddicoat, I; Afzal, NA; Hayen, A; Beattie, RM | Pharmacological treatment of gastro‐oesophageal reflux in children | 2023 | 8 | <https://www.cochranelibrary.com/cdsr/doi/10.1002/14651858.CD008550.pub3> |
| Setthawong, V; Srisubat, A; Potisat, S; Lojanapiwat, B; Pattanittum, P | Extracorporeal shock wave lithotripsy (ESWL) versus percutaneous nephrolithotomy (PCNL) or retrograde intrarenal surgery (RIRS) for kidney stones | 2023 | 8 | <https://www.cochranelibrary.com/cdsr/doi/10.1002/14651858.CD007044.pub4> |
| Virgili, G; Curran, K; Lucenteforte, E; Peto, T; Parravano, M | Anti‐vascular endothelial growth factor for diabetic macular oedema: a network meta‐analysis | 2023 | 6 | <https://www.cochranelibrary.com/cdsr/doi/10.1002/14651858.CD007419.pub7> |
| Dervenis, P; Dervenis, N; Smith, JM; Steel, DHW | Anti‐vascular endothelial growth factors in combination with vitrectomy for complications of proliferative diabetic retinopathy | 2023 | 5 | <https://www.cochranelibrary.com/cdsr/doi/10.1002/14651858.CD008214.pub4> |
| Tamminga, SJ; Emal, LM; Boschman, JS; Levasseur, A; Thota, A; Ruotsalainen, JH; Schelvis, RMC; Nieuwenhuijsen, K; van der Molen, HF | Individual‐level interventions for reducing occupational stress in healthcare workers | 2023 | 5 | https://www.cochranelibrary.com/cdsr/doi/10.1002/14651858.CD002892.pub6 |
| Iannizzi, C; Chai, KL; Piechotta, V; Valk, SJ; Kimber, C; Monsef, I; Wood, EM; Lamikanra, AA; Roberts, DJ; McQuilten, Z; So-Osman, C; Jindal, A; Cryns, N; Estcourt, LJ; Kreuzberger, N; Skoetz, N | Convalescent plasma for people with COVID‐19: a living systematic review | 2023 | 5 | https://www.cochranelibrary.com/cdsr/doi/10.1002/14651858.CD013600.pub6 |
| Stoniute, A; Madhuvrata, P; Still, M; Barron-Millar, E; Nabi, G; Omar, MI | Oral anticholinergic drugs versus placebo or no treatment for managing overactive bladder syndrome in adults | 2023 | 5 | <https://www.cochranelibrary.com/cdsr/doi/10.1002/14651858.CD003781.pub3> |
| Zhang, L; Mendoza-Sassi, RA; Wainwright, CE; Aregbesola, A; Klassen, TP | Nebulised hypertonic saline solution for acute bronchiolitis in infants | 2023 | 4 | https://www.cochranelibrary.com/cdsr/doi/10.1002/14651858.CD006458.pub5 |
| Onland, W; van de Loo, M; Offringa, M; van Kaam, A | Systemic corticosteroid regimens for prevention of bronchopulmonary dysplasia in preterm infants | 2023 | 3 | https://www.cochranelibrary.com/cdsr/doi/10.1002/14651858.CD010941.pub3 |
| Olsen, O; Clausen, JA | Planned hospital birth compared with planned home birth for pregnant women at low risk of complications | 2023 | 3 | https://www.cochranelibrary.com/cdsr/doi/10.1002/14651858.CD000352.pub3 |
| Webster, KE; Lee, A; Galbraith, K; Harrington-Benton, NA; Judd, O; Kaski, D; Maarsingh, OR; MacKeith, S; Ray, J; Van Vugt, VA; Westerberg, B; Burton, MJ | Intratympanic corticosteroids for Ménière’s disease | 2023 | 2 | <https://www.cochranelibrary.com/cdsr/doi/10.1002/14651858.CD015245.pub2> |
| Wilson, LM; Saldanha, IJ; Robinson, KA | Active cycle of breathing technique for cystic fibrosis | 2023 | 2 | https://www.cochranelibrary.com/cdsr/doi/10.1002/14651858.CD007862.pub5 |
| Gorry, C; McCullagh, L; O'Donnell, H; Barrett, S; Schmitz, S; Barry, M; Curtin, K; Beausang, E; Barry, R; Coyne, I | Neoadjuvant treatment for stage III and IV cutaneous melanoma | 2023 | 1 | <https://www.cochranelibrary.com/cdsr/doi/10.1002/14651858.CD012974.pub2> |
| Liang, F; Liu, S; Liu, G; Liu, H; Wang, Q; Song, B; Yao, L | Remote ischaemic preconditioning versus no remote ischaemic preconditioning for vascular and endovascular surgical procedures | 2023 | 1 | <https://www.cochranelibrary.com/cdsr/doi/10.1002/14651858.CD008472.pub3> |
| Ammous, O; Feki, W; Lotfi, T; Khamis, AM; Gosselink, R; Rebai, A; Kammoun, S | Inspiratory muscle training, with or without concomitant pulmonary rehabilitation, for chronic obstructive pulmonary disease (COPD) | 2023 | 1 | <https://www.cochranelibrary.com/cdsr/doi/10.1002/14651858.CD013778.pub2> |
| Jiang, S; Fang, J; Li, W | Protein restriction for diabetic kidney disease | 2023 | 1 | https://www.cochranelibrary.com/cdsr/doi/10.1002/14651858.CD014906.pub2 |
| Onland, W; Offringa, M; van Kaam, A | Late (≥ 7 days) inhaled corticosteroids to reduce bronchopulmonary dysplasia in preterm infants | 2022 | 12 | https://www.cochranelibrary.com/cdsr/doi/10.1002/14651858.CD002311.pub5 |
| Fraser, A; Poole, P | Immunostimulants versus placebo for preventing exacerbations in adults with chronic bronchitis or chronic obstructive pulmonary disease | 2022 | 11 | https://www.cochranelibrary.com/cdsr/doi/10.1002/14651858.CD013343.pub2 |
| Rodolico, A; Siafis, S; Bighelli, I; Samara, MT; Hansen, W-P; Salomone, S; Aguglia, E; Cutrufelli, P; Bauer, I; Baeckers, L; Leucht, S | Antipsychotic dose reduction compared to dose continuation for people with schizophrenia | 2022 | 11 | https://www.cochranelibrary.com/cdsr/doi/10.1002/14651858.CD014384.pub2 |
| Sereda, M; Xia, J; Scutt, P; Hilton, MP; El Refaie, A; Hoare, DJ | Ginkgo biloba for tinnitus | 2022 | 11 | https://www.cochranelibrary.com/cdsr/doi/10.1002/14651858.CD013514.pub2 |
| Smith, S; Rowbotham, NJ | Inhaled anti‐pseudomonal antibiotics for long‐term therapy in cystic fibrosis | 2022 | 11 | https://www.cochranelibrary.com/cdsr/doi/10.1002/14651858.CD001021.pub4 |
| French, HP; Abbott, JH; Galvin, R | Adjunctive therapies in addition to land‐based exercise therapy for osteoarthritis of the hip or knee | 2022 | 10 | <https://www.cochranelibrary.com/cdsr/doi/10.1002/14651858.CD011915.pub2> |
| Wilson, A; Hodgetts-Morton, VA; Marson, EJ; Markland, AD; Larkai, E; Papadopoulou, A; Coomarasamy, A; Tobias, A; Chou, D; Oladapo, OT; Price, MJ; Morris, K; Gallos, ID | Tocolytics for delaying preterm birth: a network meta‐analysis (0924) | 2022 | 8 | <https://www.cochranelibrary.com/cdsr/doi/10.1002/14651858.CD014978.pub2> |
| Zhao, Y; Dong, BR; Hao, Q | Probiotics for preventing acute upper respiratory tract infections | 2022 | 8 | https://www.cochranelibrary.com/cdsr/doi/10.1002/14651858.CD006895.pub4 |
| Wu, AD; Lindson, N; Hartmann-Boyce, J; Wahedi, A; Hajizadeh, A; Theodoulou, A; Thomas, ET; Lee, C; Aveyard, P | Smoking cessation for secondary prevention of cardiovascular disease | 2022 | 8 | https://www.cochranelibrary.com/cdsr/doi/10.1002/14651858.CD014936.pub2 |
| Flumignan, CDQ; Nakano, LCU; Baptista-Silva, JCC; Flumignan, RLG | Antiplatelet agents for the treatment of deep venous thrombosis | 2022 | 7 | <https://www.cochranelibrary.com/cdsr/doi/10.1002/14651858.CD012369.pub2> |
| Guaiana, G; Abbatecola, M; Aali, G; Tarantino, F; Ebuenyi, ID; Lucarini, V; Li, W; Zhang, C; Pinto, A | Cognitive behavioural therapy (group) for schizophrenia | 2022 | 7 | https://www.cochranelibrary.com/cdsr/doi/10.1002/14651858.CD009608.pub2 |
| López-Briz, E; Ruiz Garcia, V; Cabello, JB; Bort-Martí, S; Carbonell Sanchis, R | Heparin versus 0.9% sodium chloride locking for prevention of occlusion in central venous catheters in adults | 2022 | 7 | https://www.cochranelibrary.com/cdsr/doi/10.1002/14651858.CD008462.pub4 |
| Marson, BA; Ikram, A; Craxford, S; Lewis, SR; Price, KR; Ollivere, BJ | Interventions for treating supracondylar elbow fractures in children | 2022 | 6 | https://www.cochranelibrary.com/cdsr/doi/10.1002/14651858.CD013609.pub2 |
| Briggs, R; McDonough, A; Ellis, G; Bennett, K; O'Neill, D; Robinson, D | Comprehensive Geriatric Assessment for community‐dwelling, high‐risk, frail, older people | 2022 | 5 | https://www.cochranelibrary.com/cdsr/doi/10.1002/14651858.CD012705.pub2 |
| de Ligny, W; Smits, RM; Mackenzie-Proctor, R; Jordan, V; Fleischer, K; de Bruin, JP; Showell, MG | Antioxidants for male subfertility | 2022 | 5 | https://www.cochranelibrary.com/cdsr/doi/10.1002/14651858.CD007411.pub5 |
| El-Nakeep, S; Shawky, A; Abbas, SF; Abdel Latif, O | Stem cell transplantation for induction of remission in medically refractory Crohn’s disease | 2022 | 5 | https://www.cochranelibrary.com/cdsr/doi/10.1002/14651858.CD013070.pub2 |
| Serednicki, WT; Wrzosek, A; Woron, J; Garlicki, J; Dobrogowski, J; Jakowicka-Wordliczek, J; Wordliczek, J; Zajaczkowska, R | Topical clonidine for neuropathic pain in adults | 2022 | 5 | https://www.cochranelibrary.com/cdsr/doi/10.1002/14651858.CD010967.pub3 |
| Yoo, HHB; Nunes-Nogueira, VS; Fortes Villas Boas, PJ; Broderick, C | Outpatient versus inpatient treatment for acute pulmonary embolism | 2022 | 5 | https://www.cochranelibrary.com/cdsr/doi/10.1002/14651858.CD010019.pub4 |
| Hansel, J; Rogers, AM; Lewis, SR; Cook, TM; Smith, AF | Videolaryngoscopy versus direct laryngoscopy for adults undergoing tracheal intubation | 2022 | 4 | https://www.cochranelibrary.com/cdsr/doi/10.1002/14651858.CD011136.pub3 |
| Chau, JP; Liu, X; Lo, SH; Chien, WT; Hui, SK; Choi, KC; Zhao, J | Perioperative enhanced recovery programmes for women with gynaecological cancers | 2022 | 3 | https://www.cochranelibrary.com/cdsr/doi/10.1002/14651858.CD008239.pub5 |
| Hai, H; Li, Z; Zhang, Z; Cheng, Y; Liu, Z; Gong, J; Deng, Y | Duct‐to‐mucosa versus other types of pancreaticojejunostomy for the prevention of postoperative pancreatic fistula following pancreaticoduodenectomy | 2022 | 3 | https://www.cochranelibrary.com/cdsr/doi/10.1002/14651858.CD013462.pub2 |
| Panebianco, M; Bresnahan, R; Marson, AG | Pregabalin add‐on for drug‐resistant focal epilepsy | 2022 | 3 | https://www.cochranelibrary.com/cdsr/doi/10.1002/14651858.CD005612.pub5 |
| Tattersall, A; Ryan, N; Wiggans, AJ; Rogozińska, E; Morrison, J | Poly(ADP‐ribose) polymerase (PARP) inhibitors for the treatment of ovarian cancer | 2022 | 2 | https://www.cochranelibrary.com/cdsr/doi/10.1002/14651858.CD007929.pub4 |
| Roberts, KE; Adsett, IT; Rickett, K; Conroy, SM; Chatfield, MD; Woodward, NE | Systemic therapies for preventing or treating aromatase inhibitor‐induced musculoskeletal symptoms in early breast cancer | 2022 | 1 | https://www.cochranelibrary.com/cdsr/doi/10.1002/14651858.CD013167.pub2 |
| Garegnani, L; Hyland, M; Roson Rodriguez, P; Escobar Liquitay, CM; Franco, JVA | Antioxidants to prevent respiratory decline in people with Duchenne muscular dystrophy and progressive respiratory decline | 2021 | 12 | <https://www.cochranelibrary.com/cdsr/doi/10.1002/14651858.CD013720.pub3> |
| Kruizinga, J; Liemburg, E; Burger, H; Cipriani, A; Geddes, J; Robertson, L; Vogelaar, B; Nolen, WA | Pharmacological treatment for psychotic depression | 2021 | 12 | <https://www.cochranelibrary.com/cdsr/doi/10.1002/14651858.CD004044.pub5> |
| O'Connell, NE; Ferraro, MC; Gibson, W; Rice, ASC; Vase, L; Coyle, D; Eccleston, C | Implanted spinal neuromodulation interventions for chronic pain in adults | 2021 | 12 | https://www.cochranelibrary.com/cdsr/doi/10.1002/14651858.CD013756.pub2 |
| Sekhar, P; Tee, QX; Ashraf, G; Trinh, D; Shachar, J; Jiang, A; Hewitt, J; Green, S; Turner, T | Mindfulness‐based psychological interventions for improving mental well‐being in medical students and junior doctors | 2021 | 12 | https://www.cochranelibrary.com/cdsr/doi/10.1002/14651858.CD013740.pub2 |
| Sood, A; Mohiyiddeen, G; Ahmad, G; Fitzgerald, C; Watson, A; Mohiyiddeen, L | Growth hormone for in vitro fertilisation (IVF) | 2021 | 11 | https://www.cochranelibrary.com/cdsr/doi/10.1002/14651858.CD000099.pub4 |
| Walton, D; Castell, H; Collie, C; Wood, GK; Sharma, M; Singh, T; Michael, BD | Antiepileptic drugs for seizure control in people with neurocysticercosis | 2021 | 11 | https://www.cochranelibrary.com/cdsr/doi/10.1002/14651858.CD009027.pub4 |
| Appleton, KM; Voyias, PD; Sallis, HM; Dawson, S; Ness, AR; Churchill, R; Perry, R | Omega‐3 fatty acids for depression in adults | 2021 | 11 | <https://www.cochranelibrary.com/cdsr/doi/10.1002/14651858.CD004692.pub5> |
| Dibben, G; Faulkner, J; Oldridge, N; Rees, K; Thompson, DR; Zwisler, A-D; Taylor, RS | Exercise‐based cardiac rehabilitation for coronary heart disease | 2021 | 11 | <https://www.cochranelibrary.com/cdsr/doi/10.1002/14651858.CD001800.pub4> |
| Doyle, LW; Cheong, JL; Hay, S; Manley, BJ; Halliday, HL | Late (≥ 7 days) systemic postnatal corticosteroids for prevention of bronchopulmonary dysplasia in preterm infants | 2021 | 11 | <https://www.cochranelibrary.com/cdsr/doi/10.1002/14651858.CD001145.pub5> |
| Doyle, LW; Cheong, JL; Hay, S; Manley, BJ; Halliday, HL; Soll, R | Early (< 7 days) systemic postnatal corticosteroids for prevention of bronchopulmonary dysplasia in preterm infants | 2021 | 10 | https://www.cochranelibrary.com/cdsr/doi/10.1002/14651858.CD001146.pub6 |
| Flumignan, RLG; Trevisani, VFM; Lopes, RD; Baptista-Silva, JCC; Flumignan, CDQ; Nakano, LCU | Ultrasound guidance for arterial (other than femoral) catheterisation in adults | 2021 | 10 | <https://www.cochranelibrary.com/cdsr/doi/10.1002/14651858.CD013585.pub2> |
| Leache, L; Gutiérrez-Valencia, M; Finizola, RM; Infante, E; Finizola, B; Pardo Pardo, J; Flores, Y; Granero, R; Arai, KJ | Pharmacotherapy for hypertension‐induced left ventricular hypertrophy | 2021 | 10 | <https://www.cochranelibrary.com/cdsr/doi/10.1002/14651858.CD012039.pub3> |
| Mikolajewska, A; Fischer, A-L; Piechotta, V; Mueller, A; Metzendorf, M-I; Becker, M; Dorando, E; Pacheco, RL; Martimbianco, ALC; Riera, R; Skoetz, N; Stegemann, M | Colchicine for the treatment of COVID‐19 | 2021 | 10 | https://www.cochranelibrary.com/cdsr/doi/10.1002/14651858.CD015045 |
| Nanda, A; Hu, J; Hodgkinson, S; Ali, S; Rainsbury, R; Roy, PG | Oncoplastic breast‐conserving surgery for women with primary breast cancer | 2021 | 10 | https://www.cochranelibrary.com/cdsr/doi/10.1002/14651858.CD013658.pub2 |
| Lowe, D; Ryan, R; Schonfeld, L; Merner, B; Walsh, L; Graham-Wisener, L; Hill, S | Effects of consumers and health providers working in partnership on health services planning, delivery and evaluation | 2021 | 9 | https://www.cochranelibrary.com/cdsr/doi/10.1002/14651858.CD013373.pub2 |
| Hayden, JA; Ellis, J; Ogilvie, R; Malmivaara, A; van Tulder, MW | Exercise therapy for chronic low back pain | 2021 | 9 | <https://www.cochranelibrary.com/cdsr/doi/10.1002/14651858.CD009790.pub2> |
| Tsujimoto, Y; Miki, S; Shimada, H; Tsujimoto, H; Yasuda, H; Kataoka, Y; Fujii, T | Non‐pharmacological interventions for preventing clotting of extracorporeal circuits during continuous renal replacement therapy | 2021 | 9 | https://www.cochranelibrary.com/cdsr/doi/10.1002/14651858.CD013330.pub2 |
| Verma, GL; Spalding, JJ; Wilkinson, MD; Hofmeyr, GJ; Vannevel, V; O'Mahony, F | Instruments for assisted vaginal birth | 2021 | 9 | https://www.cochranelibrary.com/cdsr/doi/10.1002/14651858.CD005455.pub3 |
| Neil-Sztramko, SE; Caldwell, H; Dobbins, M | School‐based physical activity programs for promoting physical activity and fitness in children and adolescents aged 6 to 18 | 2021 | 9 | https://www.cochranelibrary.com/cdsr/doi/10.1002/14651858.CD007651.pub3 |
| Gianola, S; Iannicelli, V; Fascio, E; Andreano, A; Li, LC; Valsecchi, MG; Moja, L; Castellini, G | Kinesio taping for rotator cuff disease | 2021 | 8 | <https://www.cochranelibrary.com/cdsr/doi/10.1002/14651858.CD012720.pub2> |
| Offringa, M; Newton, R; Nevitt, SJ; Vraka, K | Prophylactic drug management for febrile seizures in children | 2021 | 6 | https://www.cochranelibrary.com/cdsr/doi/10.1002/14651858.CD003031.pub4 |
| Whing, J; Nandhra, S; Nesbitt, C; Stansby, G | Interventions for great saphenous vein incompetence | 2021 | 8 | https://www.cochranelibrary.com/cdsr/doi/10.1002/14651858.CD005624.pub4 |
| Hara, T; Hijikata, Y; Matsubara, Y; Watanabe, N | Pharmacological interventions versus placebo, no treatment or usual care for osteoporosis in people with chronic kidney disease stages 3‐5D | 2021 | 7 | <https://www.cochranelibrary.com/cdsr/doi/10.1002/14651858.CD013424.pub2> |
| Kerr, RS; Kumar, N; Williams, MJ; Cuthbert, A; Aflaifel, N; Haas, DM; Weeks, AD | Low‐dose oral misoprostol for induction of labour | 2021 | 6 | <https://www.cochranelibrary.com/cdsr/doi/10.1002/14651858.CD014484> |
| Brown, T; Forster, RB; Cleanthis, M; Mikhailidis, DP; Stansby, G; Stewart, M | Cilostazol for intermittent claudication | 2021 | 6 | <https://www.cochranelibrary.com/cdsr/doi/10.1002/14651858.CD003748.pub5> |
| Martin, N; Manoharan, K; Davies, C; Lumbers, RT | Beta‐blockers and inhibitors of the renin‐angiotensin aldosterone system for chronic heart failure with preserved ejection fraction | 2021 | 5 | https://www.cochranelibrary.com/cdsr/doi/10.1002/14651858.CD012721.pub3 |
| Griffiths, JD; Gyte, GML; Popham, PA; Williams, K; Paranjothy, S; Broughton, HK; Brown, HC; Thomas, J | Interventions for preventing nausea and vomiting in women undergoing regional anaesthesia for caesarean section | 2021 | 5 | <https://www.cochranelibrary.com/cdsr/doi/10.1002/14651858.CD007579.pub3> |
| Witt, KG; Hetrick, SE; Rajaram, G; Hazell, P; Taylor Salisbury, TL; Townsend, E; Hawton, K | Psychosocial interventions for self‐harm in adults | 2021 | 4 | https://www.cochranelibrary.com/cdsr/doi/10.1002/14651858.CD013668.pub2 |
| Lacey, L; Hassan, S; Franik, S; Seif, MW; Akhtar, MA | Assisted hatching on assisted conception (in vitro fertilisation (IVF) and intracytoplasmic sperm injection (ICSI)) | 2021 | 3 | <https://www.cochranelibrary.com/cdsr/doi/10.1002/14651858.CD001894.pub6> |
| Liu, C; Chen, J; Gao, Y; Deng, B; Liu, K | Endothelin receptor antagonists for pulmonary arterial hypertension | 2021 | 3 | <https://www.cochranelibrary.com/cdsr/doi/10.1002/14651858.CD004434.pub6> |
| Taylor, GMJ; Lindson, N; Farley, A; Leinberger-Jabari, A; Sawyer, K; te Water Naudé, R; Theodoulou, A; King, N; Burke, C; Aveyard, P | Smoking cessation for improving mental health | 2021 | 3 | https://www.cochranelibrary.com/cdsr/doi/10.1002/14651858.CD013522.pub2 |
| van Driel, ML; De Sutter, AIM; Thorning, S; Christiaens, T | Different antibiotic treatments for group A streptococcal pharyngitis | 2021 | 3 | https://www.cochranelibrary.com/cdsr/doi/10.1002/14651858.CD004406.pub5 |
| Gilligan, C; Powell, M; Lynagh, MC; Ward, BM; Lonsdale, C; Harvey, P; James, EL; Rich, D; Dewi, SP; Nepal, S; Croft, HA; Silverman, J | Interventions for improving medical students' interpersonal communication in medical consultations | 2021 | 2 | <https://www.cochranelibrary.com/cdsr/doi/10.1002/14651858.CD012418.pub2> |
| Minozzi, S; Pifferi, S; Brazzi, L; Pecoraro, V; Montrucchio, G; D'Amico, R | Topical antibiotic prophylaxis to reduce respiratory tract infections and mortality in adults receiving mechanical ventilation | 2021 | 1 | https://www.cochranelibrary.com/cdsr/doi/10.1002/14651858.CD000022.pub4 |
| Wuytack, F; Smith, V; Cleary, BJ | Oral non‐steroidal anti‐inflammatory drugs (single dose) for perineal pain in the early postpartum period | 2021 | 1 | https://www.cochranelibrary.com/cdsr/doi/10.1002/14651858.CD011352.pub3 |
